# Synthesis of Biocompatible Hydroxyapatite Using Chitosan Oligosaccharide as a Template

**DOI:** 10.3390/ma8125440

**Published:** 2015-11-30

**Authors:** Jinyu Wang, Guanxiong Liu, Jinshuai Chen, Bo Zhao, Peizhi Zhu

**Affiliations:** 1 School of Chemistry and Chemical Engineering, Yangzhou University, Yangzhou 225002, China; jinywang2@gmail.com (J.W.); liugangx130@gmail.com (G.L.); chenjsu7@gmail.com (J.C.); 2 Jiangsu Collaborative Innovation Center of Biomedical Functional Materials and Jiangsu Key Laboratory of Biofunctional Materials, School of Chemistry and Materials Science, Nanjing Normal University, Nanjing 210023, China; zhaobo@njnu.edu.cn

**Keywords:** chitosan oligosaccharide, hydroxyapatite, cytotoxicity, MG-63 cell

## Abstract

In this study, a novel biocompatible hydroxyapatite (HA) was synthesized by using chitosan oligosaccharide (COS) as a template. These HA samples were studied by Fourier transform infrared (FTIR) spectroscopy, X-ray diffraction (XRD), X-ray photoelectron spectroscopy (XPS), and transmission electron microscopy (TEM). The biocompatibility of HA samples was evaluated via cell viability, cell morphology and alkaline phosphatase staining of MG-63 cell lines. The results show that HA synthesized in the presence of COS was favorable to proliferation and osteogenic differentiation of MG-63 cells. These hydroxyapatites are potentially attractive biomaterials for bone tissue engineering applications.

## 1. Introduction

Over recent years, various biomaterials have been developed for bone tissue engineering applications such as bone graft substitutes, bone repair, biodegradable bone scaffold and bone drug carriers [1,2,3,4]. With stricter biocompatibility requirements for materials used in clinic application, biomimetic synthesis of hydroxyapatites with improved biocompatibility is a promising strategy for the clinic application of hydroxyapatites for bone tissue engineering [5,6,7]. The biomimetic method typically uses a bottom-up approach to design and assemble molecules into a higher order of hierarchy structure [8]. Biomolecules including polysaccharides [6,9,10], collagens [11,12], gelatin [13,14] and peptides [15,16,17] have attracted extensive attention in regulating HA nucleation and growth during the mineralization process. Wang *et al.* [11] demonstrated that the nucleation, growth, orientation and structure of bone apatite crystals was predominantly controlled by collagen matrix. Recently, much attention has been focused on the polysaccharide in the bone. Several polysaccharides including maleic chitosan, hyaluronic acid and heparin have been found to play important roles in stabilizing the amorphous calcium phosphate at the early stage of mineralization and regulating the morphology, size and crystallinity of the inorganic apatites [6,9,10]. Wise *et al.* [18] recently suggested that polysaccharides, not proteins, predominantly form an organic–mineral interface. Unlike hydrophobic collagens, the functional groups of polysaccharides can chelate Ca^2+^ ions and form hydrogen bonds with protonated PO_4_^3−^ and H_2_O on the surface of the bone apatites [19].

Chitosan, a linear polysaccharide composed of d-glucosamine and *N*-acetyl-d-glucosamine, is produced commercially by deacetylation of chitin of crustaceans such as crabs and shrimp. As the degraded products of chitosan by enzymatic and acidic hydrolysis, chitosan oligosaccharides (COS) have a lower molecular weight and are readily soluble in aqueous solutions [20]. COS have attracted considerable attention due to their biological activities and biomedical properties including antimicrobial activities, immune-enhancing effects, and anti-tumor activities [21,22,23].

As a prototype of human bone cells, the osteoblast cell line MG63 have been widely used to evaluate biocompatibility and osteogenic differentiation of materials. The sequential expression of genes for collagen type I and alkaline phosphatase (ALP) is characteristic of osteoblast differentiation of MG-63 cells [24]. In this study, we hypothesize that the amino groups play an important role in regulating the mineralization of apatites, because they have a high affinity for calcium ions in solution. The purpose of this study was to address the effects of the molecular weight and the concentration of COS on the chemical compositions, morphology and biological properties of apatites synthesized by the chemical precipitation method. All products were characterized with Fourier-transform infrared spectroscopy (FTIR), X-ray diffraction (XRD), X-ray photoelectron Spectroscopy (XPS) spectroscopy (XPS), and transmission electron microscopy (TEM). The effects of obtained HA samples on the viability and osteogenic differentiation of MG-63 cells have been evaluated by MTT assay and alkaline phosphatase staining.

## 2. Materials and Methods

### 2.1. Materials

Ca(NO_3_)_2_·4H_2_O (AR, Shanghai Chemical Reagent Co., Ltd., Shanghai, China), (NH_4_)_2_HPO_4_ (AR, Shanghai Chemical Reagent Co., Ltd., Shanghai, China), NH_3_·H_2_O (AR, Shanghai Chemical Reagent Co., Ltd., Shanghai, China), and chitosan oligosaccharide (AR, Linovus Technology, Singapore) were used as received without further purification.

### 2.2. Samples Preparation

In the typical synthesis of HA, Ca(NO_3_)_2_·4H_2_O and (NH_4_)_2_HPO_4_ were used as the calcium source and phosphorus source, respectively. The different molecular weights of COS were served as soft templates for HA synthesis. All the chemicals were used as received without further purification. The specific synthetic condition of each sample is listed in Table 1. A solution of (NH_4_)_2_HPO_4_ and COS was prepared in deionized water with magnetic stirring, then the mixed solution was heated at 85 °C. Then Ca(NO_3_)_2_ solution was added dropwisely to the heated solution, meanwhile keeping pH about 10 by adding ammonium hydroxide solution. After 2 h stirring, the hydroxyapatite suspension was aged for 24 h. The obtained precipitate was washed by deionized water and ethyl alcohol for three times, and dried with an infrared heat lamp.

**Table 1:** Synthesizing conditions for preparing apatites with COS as a template.

Sample Name	COS (g/L)	Molecular Mass of COS	Ca(NO_3_)_2_ (mol/L)	(NH_4_)_2_HPO_4_ (mol/L)	T (°C)
CS1000-1	10	1000	0.167	0.1	85
CS3000-1	10	3000	0.167	0.1	85
CS5000-1	10	5000	0.167	0.1	85
CS1000-2	2	1000	0.167	0.1	85
CS3000-2	2	3000	0.167	0.1	85
CS5000-2	2	5000	0.167	0.1	85

### 2.3. Physical Characterization Methods

#### 2.3.1. X-ray Diffraction Measurement

The crystalline phase of the samples was examined by X-ray diffraction (XRD, D8 ADVANCE, Bruker-AXS, Karlsruhe, Germany) with graphite monochromatized Cu Kα radiation operating at 40 kV and 40 mA at room temperature.

#### 2.3.2. Fourier Transform Infrared Spectrometry Measurement

Fourier transform infrared spectrometry (FTIR, ALPHA, BRUKER, Billerica, MA, USA) was used to identify the molecular structure characteristics and, after the sample stage was cleaned up by ethanol wiping, the background was tested from 500 to 3600 cm^−1^.

#### 2.3.3. X-ray Photoelectron Spectroscopy

The elements composition and the atom molar ratio of Ca to P of the samples were analyzed by X-ray photo-electronic spectroscopy (XPS, ESCALAB250Xi, ThermoFisher Scientific, Waltham, MA, USA).

#### 2.3.4. Tecnai F30 Transmission Electron Microscope Measurement

High resolution transmission electron microscopy (HRTEM, Tecnai C2 F30 S-Twin, FEI, Hillsboro, OR, USA) was carried out to determine particle size and morphology.

### 2.4. Cell Viability Test, Cell Morphology and Alkaline Phosphatase Staining Assay

Human osteosarcoma cell line MG63 cells (American Type Culture Collection, Manassas, VA, USA) were cultured in RPMI 1640 medium (Gibco, Carlsbad, CA, USA) containing 10% fetal calf serum (Gibco, Carlsbad, CA, USA), 100 μg/mL penicillin and 100 μg/mL streptomycin in a humidified atmosphere with 5% CO_2_ at 37 °C. Then MG-63 cells were seeded in a 96-well cell culture plate with a density of 5 × 10^3^ per well. The next day, cells were treated with HA samples at the concentration of 60 μg/mL (6 μg per well). After 1, 3 and 5 days, the cell viability was evaluated by MTT. Optical microscopy (Olympus BX 51M, Monolith, Japan) was used to observe the morphology of cells.

Alkaline phosphatase staining was performed with nitro blue tetrazolium/5-bromo-4-chloro-indolyl-phosphate (BCIP/NBT) Alkaline Phosphatase Color Development Kit (Beyotime Institute of Biotechnology, Nanjing, China) according to manufacturer’s instructions. Briefly, MG-63 cells were seeded in a 24-well cell culture plate with a density of 2 × 10^4^ per well. The next day, cells were treated with the HA samples with the concentration of 60 μg/mL (30 μg per well). After co-culture with samples for 4 days, the cells were washed by PBS for three times and fixed by 4% paraformaldehyde for 20 min. Then the cells were washed with PBS again for three times and incubated with BCIP/NBT staining buffer for 16 h at room temperature. The stained cells were observed under inverted microscope.

## 3. Results and Discussion

### 3.1. Fourier Transform Infrared Spectroscopy

**Figure 1:**
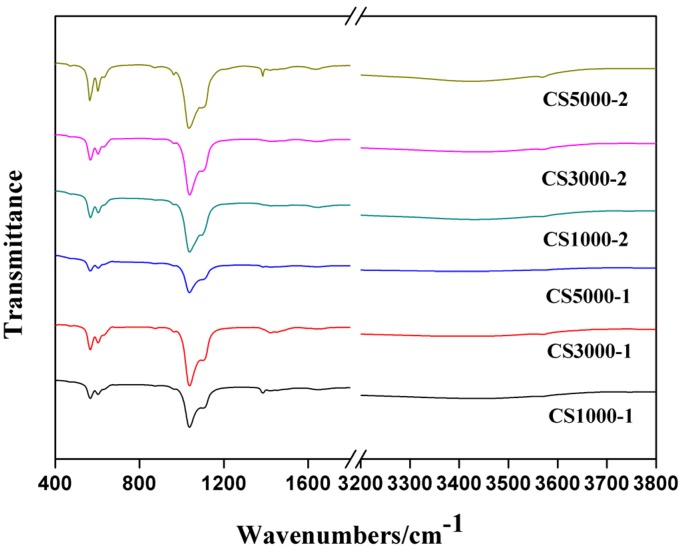
FTIR spectra of HA samples synthesized under different conditions.

The FTIR spectra of all as-prepared HA samples are shown in Figure 1. The absorption peak around 566 cm^−1^ and 603 cm^−1^ can be assigned as the bending vibration of PO_4_^3−^ [6,7,10]. The 913 cm^−1^ peak corresponds to the symmetric stretching vibration of PO_4_^3−^. The peaks at 1036 cm^−1^ and 1122 cm^−1^ should be assigned to asymmetric stretching vibration of PO_4_^3−^ [6,10]. The absorption peak around 1385 cm^−1^ belongs to CO_3_^2−^ asymmetric stretching vibration, which indicated that there is a partial substitution of CO3^2−^ for PO_4_^3−^ (B-type) [7]. The substitution of carbonate for both PO_4_^3−^ in HA was affected by the different concentration and molecular weight of COS.

### 3.2. X-Ray Photoelectron Spectroscopy

**Figure 2:**
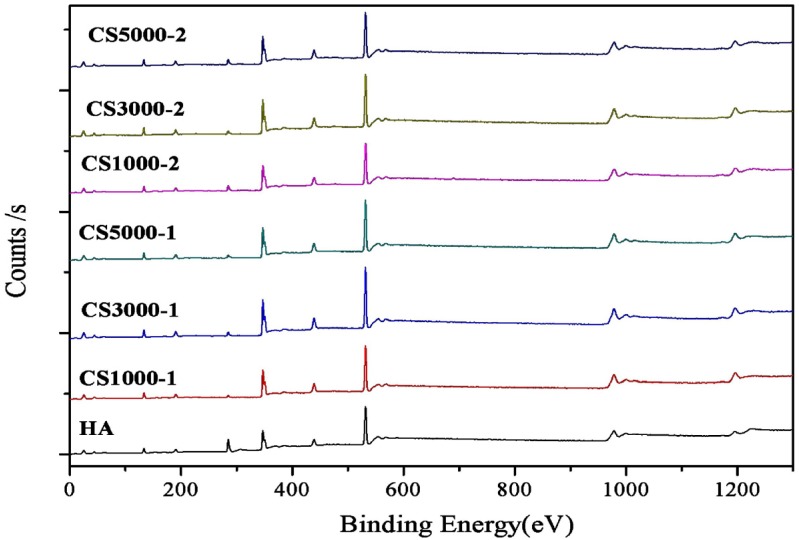
XPS spectra of as-synthesized HA samples under different conditions.

XPS characterization (Figure 2) was used to analyze the Ca/P ratio of synthesized apatite samples [25,26,27]. As shown in Table 2, the ratio between calcium and phosphate is still less than 1.67 in theory, which may be caused by the calcium deficiency on the surface of the HA lattice [13]. The ratio between calcium and phosphate of synthetic samples has no significant differences in a reasonable range. Finally, the characteristic peaks of the N element in the XPS analysis cannot be seen, which indicates that COS had been washed cleanly.

**Table 2:** Quantities and molar ratios of synthesized HA samples.

wt %	CS1000-1	CS3000-1	CS5000-1	CS1000-2	CS3000-2	CS5000-2
Ca (atomic %)	18.25	18.04	17.59	16.45	18.43	16.76
P (atomic %)	12.60	12.59	11.93	11.63	12.59	11.57
Ca/P ratio	1.45	1.43	1.47	1.41	1.46	1.45

### 3.3. X-Ray Diffraction Studies

**Figure 3:**
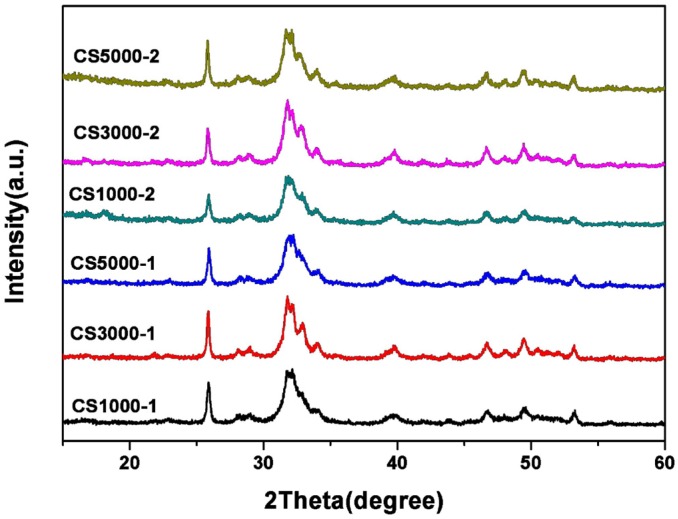
XRD patterns of as-prepared HA samples under different conditions.

XRD patterns of the six synthesized HA samples are shown in Figure 3. The diffraction peaks of all HA samples agree with those of pure HA at 2θ values of 25.9°, 31.7°, 32.9°, 39.8°, 46.7°, 49.5° and 53.1°, which are indexed to (002), (211), (300), (310), (222), (213) and (004) planes, respectively [6,7,10]. The intense bands at around 2θ = 26° and 2θ = 33° prove that the samples are predominantly HA. The patterns of HA samples with broader bands are similar to those minerals in human bones [28]. The amino groups on the surface of COS can chelate Ca^2+^ ions to form ionic clusters for the nucleation of minerals and function like a natural template for the apatite crystal to grow.

### 3.4. Morphology Analysis Using TEM

**Figure 4:**
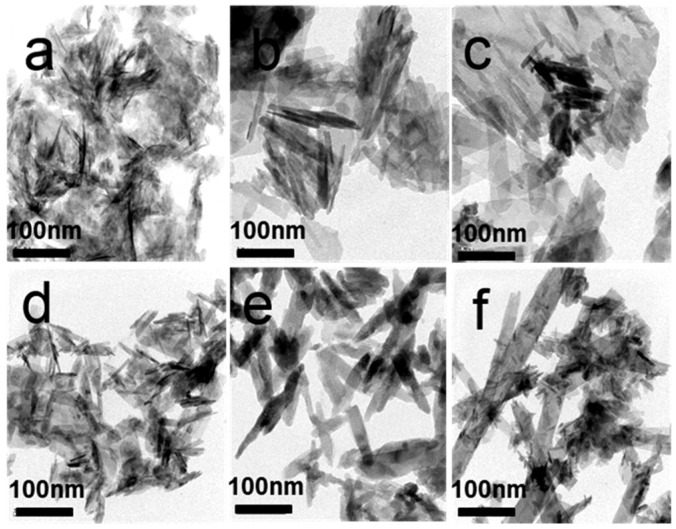
TEM micrographs of HA samples: (**a**) CS1000-1; (**b**) CS3000-1; (**c**) CS5000-1; (**d**) CS1000-2; (**e**) CS3000-2; (**f**) CS5000-2.

Figure 4 shows TEM micrographs of the as-synthesized samples. The HA samples COS1000-1 and COS 1000-2 synthesized by using COS of 1000 molecular weight (MW) as a template featured very irregular needle-like morphology (Figure 4a,e). The crystal structure of the HA samples COS3000-1 and COS 3000-2 synthesized with 3000 molecular weight (MW) COS is close to the irregular nanorods (Figure 4b,f). With the increase of the COS concentration, the morphology and size of apatites crystals show few changes. However, as the molecular weight of COS increase, the size of the HA crystals also visibly increase. This indicates that the presence of COS in solution may interfere with the growth of HA crystals. Longer chains of larger COS molecules chelate with calcium ions on the surface of apatite crystals, function as templates to attract calcium ions to promote nucleation of minerals and guide the growth of crystals along the chains.

### 3.5. Cell Viability Test, Cell Morphology and Alkaline Phosphatase Staining Assay

**Figure 5:**
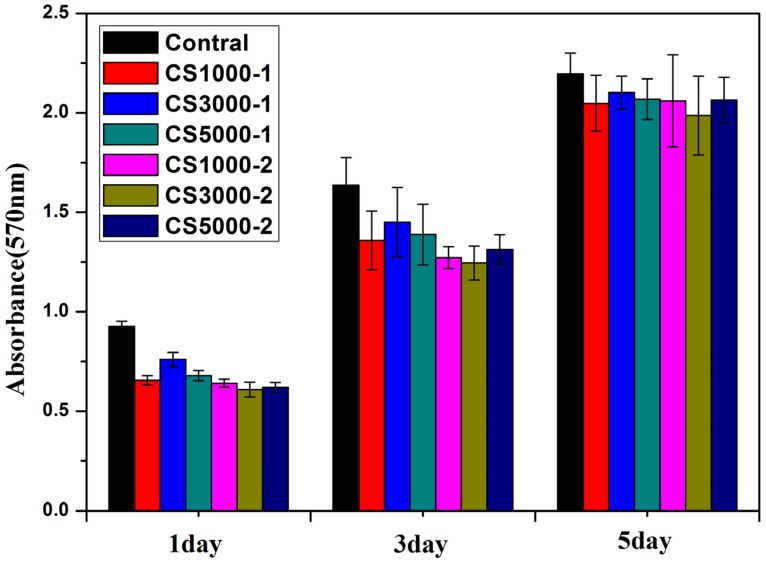
*In vitro* cytotoxicity of MG-63 cell lines after culturing with HA samples.

**Figure 6:**
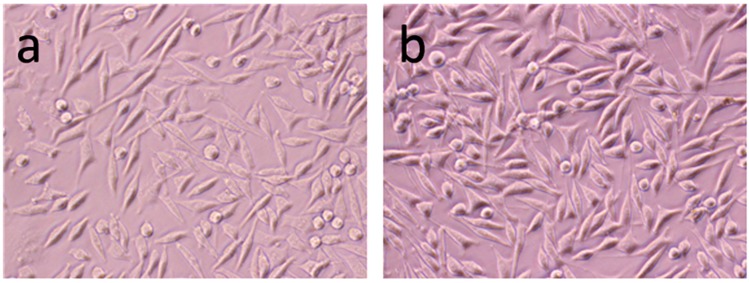
Cell topography of MG-63 cells co-cultured with 60 ug/mL of COS3000-1 sample for different times: (**a**) 24 h; (**b**) 72 h.

The *in vitro* biocompatibility of HA samples was assessed by MTT assay on an MG-63 cell line. The MG-63 cells were co-cultured with HA samples for 1 day, 3 days and 5 days. As shown in Figure 5, after 1 day culturing, the cell viability after culturing with HA samples were slightly lower than control group, which could be explained by the cytotoxicity of HA nanoparticles. Although HA has been widely used as biomaterials for tissue engineering and drug carriers, it has been reported that hydroxyapatite nanoparticles induce apoptosis on MC3T3-E1 cells and tissue cells in SD rats [29]. The cytotoxicity of HA nanoparticles is mostly dependent on its shape and cell types [30]. After 1 day culturing, the cell viability of COS3000-1 was slightly higher than other group, indicating that the molecular weight and concentration might have an impact on biocompatibility of HA samples. From Figure 5, it can be seen that the viability of MG-63 cells incubated with each sample (60 ug/mL) for 5 days still displays few differences between each other, except that COS3000-1 shows a bit better vitality. The cell morphology of MG-63 cells co-cultured with COS3000-1 was shown in Figure 6. The Figure 6a shows the cell morphology images observed from MG-63 cells cultured with HA samples at 60 μg/mL concentration for 1 day. The MG63 looked natural, attached and well-spread on the dish surface. Figure 6b shows that MG-63 cells became extremely dense after 72 h co-culturing with 60 ug/mL of COS3000-1.

The distinct 570 nm absorbance for MTT could be ascribed to the difference in the morphology, carbonate content and the Ca/P ratio of the synthesized HA crystals. The substitution of carbonate of HA can enhance its solubility, which makes HA biologically active [6,10]. In addition, calcium-deficient apatites are also of biological importance since the catalytic activity of HA is proportional to the calcium deficiency of the sample. Large amounts of carbon, non-stoichiometric Ca/P ratio and appropriate nano-morphology may be the co-contributors to the biocompatibility of apatites [31,32]. After 3 days incubation, all HA samples only show lower absorbance than the control group, but little difference between each other. Similar results are obtained after 5 days incubation. This can be explained by little structural difference between each sample characterized by FTIR, XPS and TEM. However, COS3000-1 displays a bit better vitality, which is mainly due to its appropriate morphology of crystalline, low Ca/P ratio as well as certain amounts of carbonate substitution. The cell vitality test shows that these HA samples are biological apatites and biocompatible with the human osteosarcoma MG-63 cell line.

**Figure 7:**
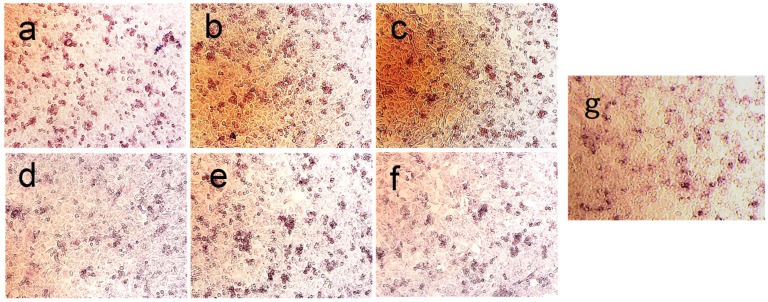
ALP activity images of MG-63 cells co-cultured with HA samples: (**a**) CS1000-1; (**b**) CS3000-1; (**c**) CS5000-1; (**d**) CS1000-2; (**e**) CS3000-2; (**f**) CS5000-2; (**g**) Control.

Alkaline phosphatase expression is indicative of osteogenesis. As shown in Figure 7, after co-culturing with 60 μg/mL concentration of HA samples for 4 days, alkaline phosphatase is expressed in large amounts in the cell cytosol of MG-63 cells. Since alkaline phosphate is expressed in large amounts in the differentiation phase of pre-osteoblastic MG63 cells, the assay is able to show early osteoblastic phenotypic expressions, which is indicative of osteogenesis [33]. The results indicate that all synthesized HA samples prepared with different molecular weights and concentrations of COS have similar impacts on the growth and osteogenic differentiation MG-63 cells. The ALP assay results demonstrate that the HA samples synthesized with a higher concentration of COS (Figure 6a–c) show better osteogenic differentiation activity.

## 4. Conclusions

In this study, chitosan oligosaccharides with different molecular weights were used as the templates to synthesize apatite samples. The effects of the chitosan oligosaccharide concentration and the molecular weight on the chemical composition, morphology, and biocompatibility of as-synthesized apatites were investigated using FTIR, XRD and TEM characterization techniques. It was found that these non-stoichiometric carbonated apatites were favorable to the proliferation and differentiation of MG-63 cells. These novel biocompatible hydroxyapatites synthesized by using chitosan oligosaccharide as a template are promising candidates for bone tissue engineering applications.
